# Pervasive Growth Reduction in Norway Spruce Forests following Wind Disturbance

**DOI:** 10.1371/journal.pone.0033301

**Published:** 2012-03-07

**Authors:** Rupert Seidl, Kristina Blennow

**Affiliations:** 1 Faculty of Landscape Planning, Horticulture and Agricultural Science, Swedish University of Agricultural Sciences, Alnarp, Sweden; 2 Institute of Silviculture, University of Natural Resources and Life Sciences (BOKU), Vienna, Austria; DOE Pacific Northwest National Laboratory, United States of America

## Abstract

**Background:**

In recent decades the frequency and severity of natural disturbances by e.g., strong winds and insect outbreaks has increased considerably in many forest ecosystems around the world. Future climate change is expected to further intensify disturbance regimes, which makes addressing disturbances in ecosystem management a top priority. As a prerequisite a broader understanding of disturbance impacts and ecosystem responses is needed. With regard to the effects of strong winds – the most detrimental disturbance agent in Europe – monitoring and management has focused on structural damage, i.e., tree mortality from uprooting and stem breakage. Effects on the functioning of trees surviving the storm (e.g., their productivity and allocation) have been rarely accounted for to date.

**Methodology/Principal Findings:**

Here we show that growth reduction was significant and pervasive in a 6.79·million hectare forest landscape in southern Sweden following the storm Gudrun (January 2005). Wind-related growth reduction in Norway spruce (*Picea abies* (L.) Karst.) forests surviving the storm exceeded 10% in the worst hit regions, and was closely related to maximum gust wind speed (R^2^ = 0.849) and structural wind damage (R^2^ = 0.782). At the landscape scale, wind-related growth reduction amounted to 3.0 million m^3^ in the three years following Gudrun. It thus exceeds secondary damage from bark beetles after Gudrun as well as the long-term average storm damage from uprooting and stem breakage in Sweden.

**Conclusions/Significance:**

We conclude that the impact of strong winds on forest ecosystems is not limited to the immediately visible area of structural damage, and call for a broader consideration of disturbance effects on ecosystem structure *and* functioning in the context of forest management and climate change mitigation.

## Introduction

Natural disturbances have been increasing in frequency and severity in forests around the world in recent decades [1], [2]. As a consequence, disturbances by e.g., wildfires, insect outbreaks, and strong wind events are increasingly becoming a challenge for the sustainable management of forest ecosystems. Disturbances can have strong negative effects on timber production and the timber-based economy, e.g., through a devaluation of wood, the need to harvest prematurely, and pulses of salvaged timber saturating the market. A single storm event on January 8^th^–9^th^ 2005 (storm “Gudrun”), for instance, was estimated to have caused an overall economic damage of 2.4 billion Euros in Swedish forestry alone [3]. In addition, disturbance events can turn forests acting as a carbon (C) sink to the atmosphere into a C source [4], [5]. They thus have the potential to strongly interfere with objectives of mitigating climate change through forest management. In turn, anthropogenic climate change is also affecting disturbance regimes. The observed intensification of disturbances has been partly linked to recent changes in the climate system [6], [7], and projections under future climate scenarios point towards a further increase in disturbances [8], [9]. Addressing disturbances is thus increasingly becoming a central issue in the sustainability sciences in general and in ecosystem management in particular [10].

**Figure 1:**
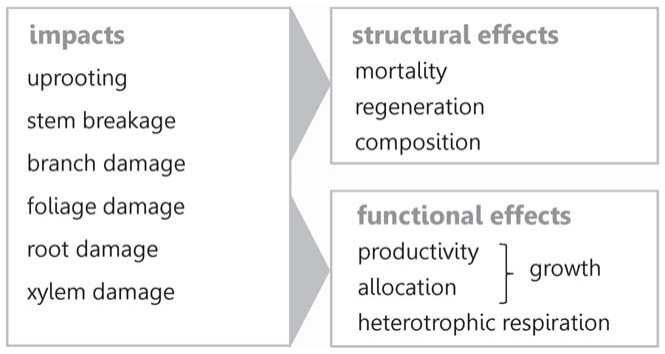
The impacts of strong winds and their effects on forest ecosystem structure and functioning. Storms can have various impacts on trees, ranging from uprooting to mechanical damage of different tree compartments. These impacts result in two different types of responses: an effect on ecosystem structure (e.g., via tree mortality), and a functional response (i.e., changes in fundamental ecophysiological processes). Structural effects relate to changes in demographics and composition, while functional effects concern the response of the existing/remaining vegetation and soil. Assessments of wind effects in the context of forestry and ecosystem management have to date focused largely on the former, while we here estimate the significance and magnitude of the functional effect of strong winds at the landscape scale, focusing on short-term tree growth changes. Note that this schematic illustration focuses on first order effects only; structural effects will influence functioning (and vice versa) over the course of forest dynamics.

Strong winds are the most important disturbance agent in European forest ecosystems (judged by the volume of timber damaged, see [1]). The impacts of wind on trees are manifold, and range from their uprooting to mechanical damage of individual tree compartments (Figure 1). Wind effects on forest structure and demography, i.e., mortality resulting from uprooting and stem breakage, have been the main concern in the context of forestry and ecosystem management to date. They are highly visible in the landscape and are of considerable magnitude, amounting to 18.7 million m^3^ per year on average over the period 1950 to 2000 in European forests, and reaching a peak annual damage of 180 million m^3^ in 1990 [1]. However, also the surviving trees and stands in landscapes affected by strong winds are impacted by the physical force of such atmospheric extremes. The ecological literature on their effects on the residual tree population reports both an increase (due to a release from wind-killed neighbors and the subsequent increase in resource availability for surviving trees, see e.g., [11]–[13]) and a decrease (due to mechanical damage to branches, roots, and xylem tissue, see e.g., [14], [15]) in growth and productivity in response to strong winds. Furthermore, mechanical stimulation from wind was found to considerably change the allocation of carbohydrates within trees, increasing the allocation to belowground compartments [16], [17]. However, these effects on fundamental ecophysiological processes in the surviving tree population, summarized as functional effects here, are to date not considered in assessments of wind damage in the context of forestry and sustainable forest management, largely because a single storm can have simultaneously positive and negative effects at the level of individual trees [18], and the overall sign and magnitude of functional effects on forest landscapes is still unresolved. Yet, ignoring such functional effects could lead to a considerably biased appraisal of wind disturbance, e.g., with regard to effects on timber production and carbon storage, in ecosystem management.

Our overall objectives were thus to investigate the surviving tree population for functional wind effects, and quantify the magnitude of these effects at the forest landscape level. Since effects on survivors' growth were widely disregarded in previous landscape scale assessments of wind impacts (e.g., [3]) we tested the hypothesis that the short-term growth of surviving forests remained unaffected by strong winds, with the alternative hypothesis that growth changed significantly in the three years following the storm. To that end we studied the 6.79·million ha forest landscape in southern Sweden that was affected by the storm Gudrun in January 2005. Judged by the structural damage of approximately 75·million m^3^ of wood (which equals 110% of the average annual harvest 1998–2004 in Sweden from only 16% of the country's forest area) Gudrun was the worst storm on record for Sweden [19]. Yet, this drastic damage accrued on less than 6% [20] of the overall area affected by the strong winds of the Gudrun weather system (for which gust speeds of up to 42 m·s^−1^ have been reported, [3]). Here we focus on the residual 94% of the landscape, i.e., on the surviving tree population, and investigate growth changes for its dominant tree species (i.e., Norway spruce, *Picea abies* (L.) Karst.) in the years 2005–2007.

## Results

### Climate response functions to control for the effect of climate variation

We applied response function analysis to remove the effect of climate variation on the observed growth time series, using monthly temperature and precipitation between June of the preceding year and September of the current year as explanatory variables. Climate factors explained 63.0% of the variance in tree growth on average over all counties (Table 1). This level of determination is well in the range reported by previous studies for Scandinavia, finding climate variation to determine between 24% and 82% of the annual variation of tree growth [21]–[25]. Over all counties, temperature and precipitation in May and June of the current year were found to have the strongest positive impact on tree increment, but the influence of individual climate drivers varied considerably between counties. Climate factors in the preceding growing season together accounted for roughly 40% of the overall climate influence on tree growth [23], a finding that was subsequently used to inform our hypothesis on the temporal pattern of functional wind effects (see Material and Methods).

**Table 1:** Model diagnostics.

	response function analysis	interrupted time series analysis	
county	coefficient of determination (dimensionless)	mean bias (%)	max. residual autocorrelation (dimensionless)
Ö-V	0.595	−0.3	−0.276
U-S-S	0.678	+0.6	0.295
Ö-K	0.411	+0.1	0.235
S-Ä	0.839	−1.3	−0.255
J-K	0.577	+0.2	−0.204
G-H	0.623	−0.7	0.169
M-K-B	0.685	+0.3	−0.326

**Figure 2:**
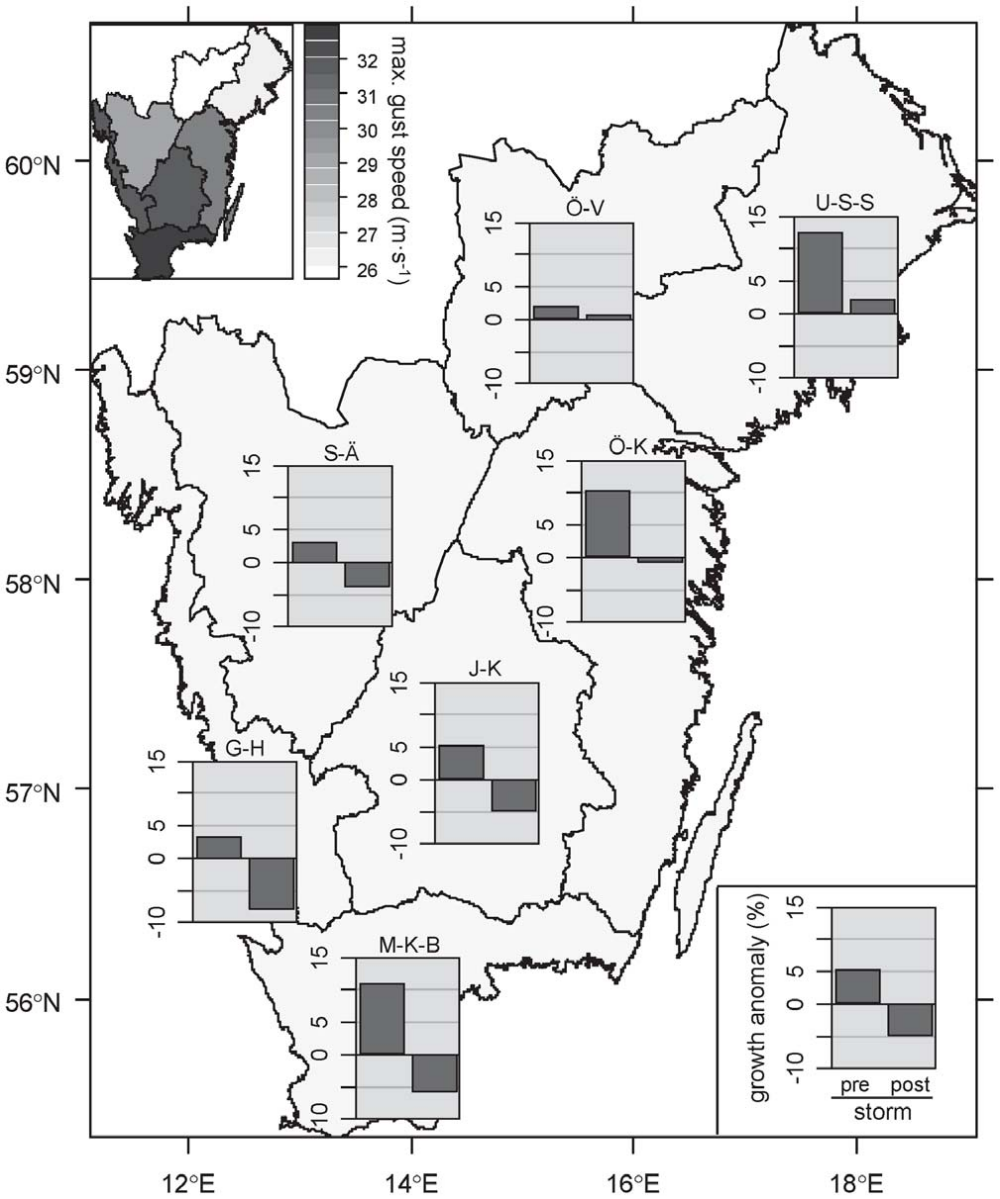
Tree growth anomalies before and after the storm Gudrun (January 2005) in southern Sweden. The depicted growth anomalies are the deviation from the long-term, age-de-trended growth series mean after controlling for the effect of climate variation. Bars indicate mean values over the three vegetation periods pre and post storm. The insert map shows area-averaged maximum gust speeds during Gudrun [3]. Spatial entities are the county-groups used by the Swedish National Forest Inventory [27]. U-S-S: Uppsala, Stockhom & Södermanland; Ö-V: Örebro & Västmanland; S-Ä: Skaraborg & Älvsborg; Ö-K: Östergötland & Kalmar; J-K: Jönköping & Kronoberg; G-H: Göteborg & Halland; M-K-B: Malmöhus, Kristianstad & Blekinge.

All diagnostics given for the interrupted time series models are not significant at α = 0.05. Significance of the residual autocorrelation was tested by means of a Box-Pierce test, and the maximum autocorrelation coefficient is reported here. For county abbreviations and location see Figure 2.

### Exploratory analysis of growth before and after the storm

**Figure 3:**
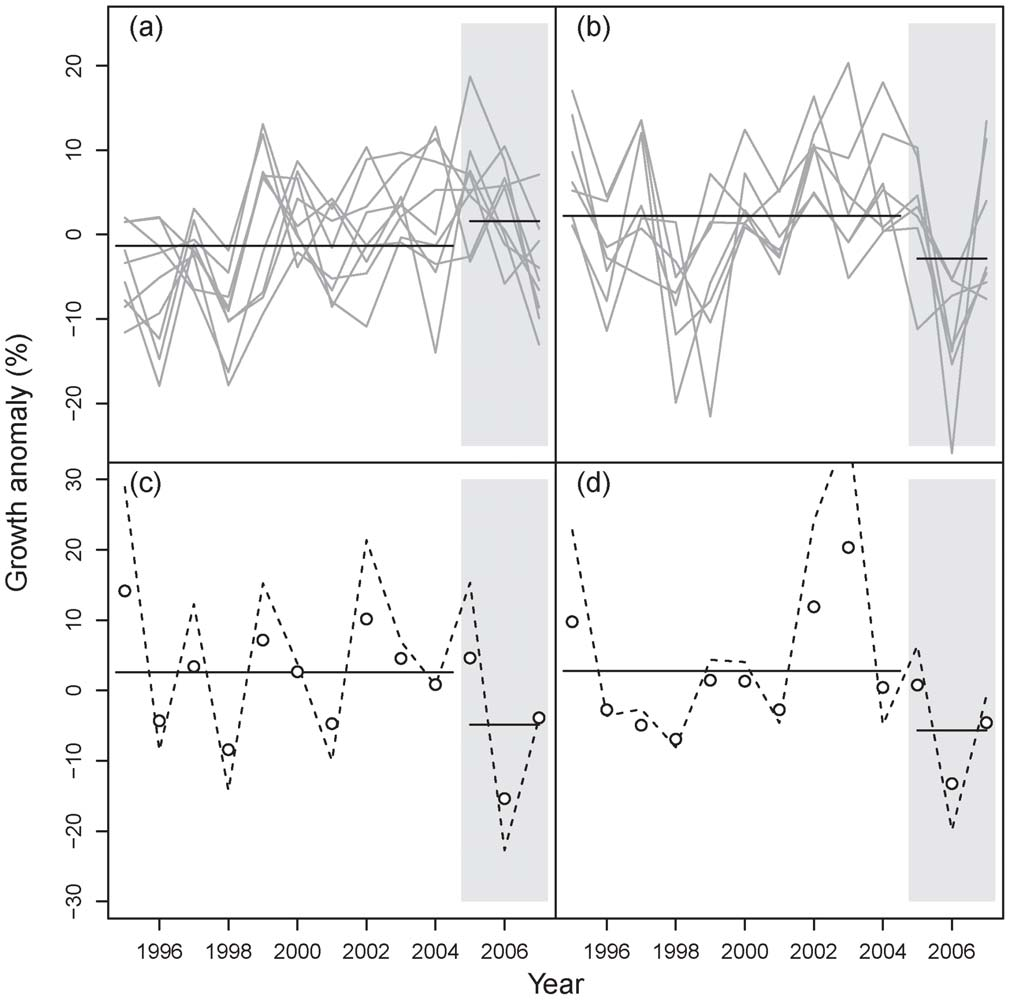
Norway spruce growth anomalies over time. The upper panels compare growth anomalies (a) for the counties not affected by Gudrun (i.e., Norrbotten-Lappmark, Norrbotten-Kustland, Västerbotten-Lappmark, Västerbotten-Kustland, Jämtland, Västernorrland, Gävle, Dalarna, Värmland) to (b) those that were hit by the storm in January 2005 (see Figure 2). The lower panels give examples of the temporal development of observed growth anomalies (circles) for the counties Jönköping & Kronoberg (c) and Malmöhus, Kristianstad & Blekinge (d) with the fitted interrupted time series models indicated as dashed line. Solid horizontal lines in all panels denote the average growth anomaly in the ten years prior to the storm and in the three years following Gudrun (grey shaded area).

After controlling for the influence of climate variability on tree growth an exploratory analysis revealed widespread growth reduction in the years following the storm (Figure 2). Norway spruce increment dropped in all counties affected by Gudrun compared to pre-storm levels. In the three years after the storm growth levels were below the long-term average in five out of the seven counties investigated (ranging from −8.0% to −0.7% below the long-term mean). These were also the counties for which the highest wind speeds were reported during Gudrun (Figure 2). Moreover, while Norway spruce growth anomalies (after controlling for the influence of climate) in the years 2005–2007 showed a positive trend for the rest of Sweden they decreased in the counties affected by Gudrun (Figure 3a,b).

### Interrupted time series analysis to test for wind effects on growth

To more systematically investigate functional wind effects and account for temporal patterns and variation in growth we conducted an interrupted time series analysis, testing for significance and magnitude of a wind-induced growth reduction in the three years after the storm. The order of the autocorrelation process (i.e., the AR component) and the moving average process (i.e., the MA component) in the final fitted models ranged between zero and two, respectively. The final ARIMA models were unbiased, and their residuals not significantly autocorrelated (Table 1). The assumed temporal perturbation pattern generally fit the observations well (see Figure 3c,d), and a systematic sensitivity analysis of perturbation dummies found the average sensitivity of the estimated perturbation coefficients over all counties to be moderate (0.3±2.0 percentage points).

We found that growth reductions were significant (α = 0.05) over the residual variation in the growth time series in the three southwestern counties of the study region (Table 2). In addition, the growth reductions in all but the northernmost counties were found to be marginally significant, which is noteworthy considering that the available time series were relatively short (n = 56) compared to the recommendations for interrupted ARIMA analysis [26]. We thus had to reject the hypothesis that Norway spruce growth was not affected by Gudrun. Estimates for the maximum annual growth reduction, i.e., the perturbation coefficients of the interrupted time series analysis, ranged from −10.6% to 0.0% (Table 2), with an average of −6.2% over the whole area affected by Gudrun (weighted by pre-storm standing volume).

**Table 2:** Structural (mortality) and functional (growth reduction) effects of the storm Gudrun.

	relative effects		absolute effects	
county	mortality(% of growing stock)	growth reduction(% of long-term mean)	mortality(10^6^ m^3^)	growth reduction(10^6^ m^3^)
Ö-V	0.4	0.0	0.69	0.00
U-S-S	1.2	2.8	0.69	0.17
Ö-K	4.3	6.4**'**	10.21	0.50
S-Ä	7.1	3.1**'**	9.45	0.26
J-K	14.3	10.4 *	38.45	1.09
G-H	11.9	10.6 *	6.15	0.55
M-K-B	7.4	8.2 *	8.96	0.45

Relative growth reductions were determined as perturbation coefficients in an interrupted time series analysis (*: significant at α = 0.05, **'**: marginally significant at α = 0.15) and are given here as the maximum annual values in the second vegetation period after the storm. Note that structural effects are reported for all species while functional effects pertain to Norway spruce only. For county abbreviations and location see Figure 2.

**Figure 4:**
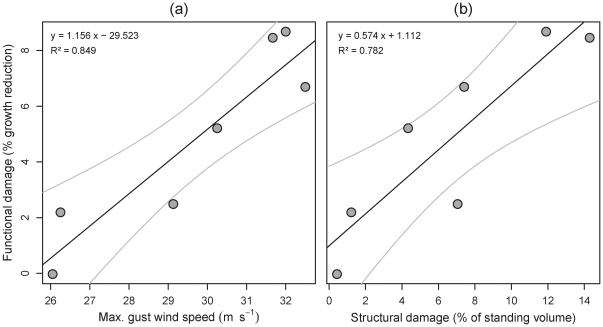
Relationship of functional wind effects to maximum gust wind speed and structural damage. The panels show growth reduction explained by maximum gust wind speed (a) and tree mortality from windthrow and –breakage (b) for the counties affected by the storm Gudrun. Functional wind effects are given as annual growth loss averaged over the first three growing seasons after the storm, and were derived as perturbation coefficients in an interrupted ARIMA analysis. Since the intercept of the regression in panel (a) was not significant, also a no-intercept model was fitted, resulting in a regression coefficient of 0.672 (P<0.001). Maximum gust wind speeds in panel (a) represent the maximum over the duration of the storm spatially averaged over the county area.

These pervasive functional effects were strongly related to the wind speeds of the Gudrun weather system (Figure 4a). A linear relationship between area-averaged maximum gust speeds and perturbation coefficients explained 85% of the variation in growth reduction, and suggests that this type of wind damage occurs if large-scale maximum gust speeds exceed 25.5 m·s^−1^. The relationship between structural and functional storm effects (Figure 4b) was slightly less strong, but damage percentage from windthrow and –breakage still explained more than three fourth of the observed variation in growth reduction after storm. Our results suggest that for every 10% of the standing timber volume structurally damaged by Gudrun a 6.7% growth reduction occurred on average in the three years following the storm.

### Quantifying functional wind effects at the landscape scale

Applying the estimated perturbation coefficients to the upscaled increment values of the Swedish National Forest Inventory [27] we estimate that the storm Gudrun caused an overall loss in Norway spruce growth of 3.0 million m^3^ between 2005 and 2007. In the counties Jönköping and Kronoberg alone this growth reduction exceeded 1 million m^3^ (Table 2). The overall growth loss in the first three years following the storm was thus larger than the average structural damage from the 77 storms on record between 1900 and 2004 for Sweden, which was calculated to 1.4 million m^3^ by Nilsson *et al.* (2004). It furthermore was in the same order of magnitude as the volume damaged by spruce bark beetle (i.e., a major secondary disturbance agent following wind damage in Norway spruce ecosystems) in southern Sweden in the four years following Gudrun [28]. We can also compare the growth losses resulting from the single storm event Gudrun to the average annual continental scale damage by bark beetles (i.e., the most detrimental biotic disturbance agent in Europe), which was estimated to amount 2.9 million m^3^ between 1950 and 2000 [1].

## Discussion

Functional disturbance effects such as growth changes after wind events have to date received only limited attention in the literature (note for instance their absence in the seminal review by Everham and Brokaw [12]). Furthermore, such effects are currently neglected in the monitoring and economic assessment of wind damage in the context of forest management, which focus exclusively on structural effects [1], [3], [29]. While both positive and negative wind-induced growth changes have been reported at the level of individual trees [11]–[18], we have shown here that the storm Gudrun resulted in a pervasive and significant growth reduction in Norway spruce forests at the landscape scale. The considerable magnitude of the growth reduction documented here highlights the importance of a more holistic consideration of disturbance-mediated changes of ecophysiological processes in assessments of disturbance effects on timber resources and forestry (see [30]).

Our findings are likely to also have implications for the forest C cycle: Lindroth et al. [5] found a profound alteration of the forest C balance at sites windthrown by the storm Gudrun. Since stem growth is closely related to net ecosystem productivity in mature forests [31], our findings suggests that C cycle effects might not be limited to sites directly damaged by the storm but might be far more widespread in landscapes affected by strong winds. However, in order to quantify these effects with fidelity an investigation into the underlying causes is required. While our data document the existence and significance of such a net wind effect at the landscape scale, the question whether it is predominantly caused by reduced primary productivity due to hampered resource utilization (resulting from mechanical damage to roots, xylem, and crown), or due to changes in allocation (prioritization of belowground compartments in response to mechanical damage and stimulation from wind, or allocation to defense compounds to ward off insect attacks) has to remain unanswered.

Results from detailed empirical experiments in neighboring Denmark suggest root damage as a plausible cause of the observed growth losses: Nielsen and Knudsen [14], conducting tree pulling and liberation experiments, found that Norway spruce trees exposed to increased mechanical force from wind suffered from a considerable loss in root anchorage tied to damage of the rooting system. Their sample trees responded with a subsequent prioritization of root growth at the cost of upper stem compartments, amounting to a five year increase in root basal area of 230%. In this regard the analysis by Vargas et al. [32] documented that trees can mobilize a significant amount of stored carbohydrates to compensate for root losses after a hurricane. The central role of root damage in the context of the growth reduction reported here is furthermore supported by evidence of increased drought sensitivity after Gudrun [33]. Empirical studies, finding a significant loss in Norway spruce root anchorage and increasing propensity for subsequent structural damage by wind following Gudrun, also report a direct correlation to indicators of tree vigor and water balance [14], [34].

Since both wind and drought disturbance have been independently predicted to increase in the future [35], [36] amplifying feedbacks between them would have the potential to contribute to a further intensification of disturbance regimes under climate change (see also [37], [38]). Furthermore, since tree growth and vigor have been related to tree defense against bark beetles [39], a decreased productivity and increased drought-proneness due to root damage could be a major mechanism driving the widely observed (e.g., [40]) increase in colonization success and mortality from bark beetles after storm.

With regard to these disturbance interactions an important limitation in our understanding of growth reductions after strong winds concerns its duration and persistence. We found significant landscape scale effects for the first three vegetation periods after Gudrun, but cannot from our data infer how long a large-scale recovery will take. Busby et al. [41], for instance, reported growth loss after Hurricane disturbance to last up to five years in individual stands. Nielsen [34] found that in two thirds of the investigated trees at least 50% of the loss of root anchorage remained three years after the storm Gudrun. Our upscaled results might thus represent a first conservative estimate of the overall magnitude of functional wind effects after Gudrun. The ability to recover to pre-disturbance growth levels is likely also dependent on the presence of additional stressors and the general health condition of the ecosystem. Furthermore, it has to be noted that we have focused solely on Norway spruce forests in our analysis, and differential effects might be expected for species with different root- or crown architecture, such as for Scots pine (*Pinus sylvestris* L.), a major associate of Norway spruce in our study region.

In conclusion, while disturbances are considerably changing in response to changing climate and management regimes [7], our understanding of their impacts on forest resources and ecosystem dynamics is still incomplete. In this regard the significant functional wind effect reported here highlights the importance of considering disturbance effects beyond immediate tree mortality in order to develop a more holistic account of how disturbances affect ecosystems [42], [43]. The latter is urgently needed as natural disturbances are increasingly challenging sustainable forest management, threatening to interfere with objectives to increase the use of renewable resources, and mitigate climate change through ecosystem management [5], [44]. Considering the magnitude of the functional effects reported here, we conclude that the impact of wind disturbance on forest resources and carbon budgets might have been underestimated previously, and call for a broader consideration of disturbance effects on ecosystem structure *and* functioning in the context of forest management and climate change mitigation.

## Materials and Methods

### Data

The response variable in our analysis was stem growth (i.e., a proxy for ecosystem functioning, Figure 1) of trees surviving the storm Gudrun, derived from tree cores taken by the Swedish National Forest Inventory (NFI). All necessary permits were obtained for the described field studies (Swedish law 2001:99). NFI systematically samples trees all over Sweden with the aim of deriving growth information representative for the full range of stand and ownership categories at the level of counties (or combinations thereof, see Figure 2). The tree rings are analyzed by NFI, i.e., cross-dated, and the age-trend removed by standard tree ring analysis procedures [45]. Since NFI revisits only a fraction of the systematically selected sample locations every year we used data from four consecutive inventory years (2007–2010) in our analysis. Overall, our analysis is based on data from 866 individual tree cores (between 59 and 181 per study entity), which were aggregated to generate representative growth time series per county for the years 1952 to 2007. Only tree cores of individuals with age >60 years were considered in the analysis. Furthermore, we focused on Norway spruce, which is the dominant species in the study area (proportion on total standing timber volume prior to the storm Gudrun: 46.6%, [27]), and was also the most affected species by structural storm damage from Gudrun [20].

To remove the effect of climate variation on our response variable (i.e., tree ring index) we conducted a response function analysis (i.e., principal component regression, see [46], [47]), using monthly temperature and precipitation as predictors. Climate data for the period 1952 to 2007 were derived from the reanalysis dataset of Kalnay et al. [48] (2.5°×2.5° spatial resolution). The monthly variables were centered and scaled with their time series mean and standard deviation prior to their use in the response function analysis. Building on previous findings for Scandinavia [21] we used the 16 months from June of the previous year to September of the current year as potential predictors for a given years' tree ring index. Orthogonality of predictors was ensured by conducting a principal component analysis [49]. We retained all principal components as predictors in the initial regression analysis [50], and subsequently chose the best and most parsimonious model by backwards selection using Akaike's information criterion (AIC) as performance indicator. The residuals of the selected model (i.e., the variation in tree ring index not attributed to variation in climate in the response function analysis) were used as response variable in the subsequent analyses of wind effects on forest growth.

### Interrupted time series analysis

After an exploratory analysis of the growth anomalies in the vegetation periods before and after the storm we conducted an interrupted time series analysis to detect whether the storm event of January 2005 significantly influenced growth in the growing seasons following it [51], [52]. We fitted autoregressive integrated moving average (ARIMA) models to the 56 year growth anomalies, using AIC for model selection. The integration parameter of the ARIMA was set to zero, since no drift was expected due to prior de-trending of the tree ring index. We subsequently included a perturbation component in the model to study the impact of Gudrun on the growth anomalies. Assuming that the temporal variation is adequately explained by the ARIMA model, a significant increase in explanatory power through the inclusion of such a perturbation component would indicate that the hypothesis of no effect of Gudrun on growth would have to be rejected [51]. The perturbation component, specified as dummy variables between 0 (i.e., no effect, in the years before Gudrun) and 1 (i.e., maximum effect), was formulated to be gradual in onset and temporary in duration. We assumed the functional wind effect to reach its maximum only in the second growing season after the storm, accounting for time lags in ecophysiological adjustments of trees (e.g., with regard to allocation priorities, see [53]) and a buffering effect of stored carbohydrates in the first growing season following the disturbance. Gough et al. [54], for instance, found that annual observations of photosynthesis and stem growth were temporally decoupled due to late season photosynthesis being allocated to stem growth in the following spring (see also [23], and our results on previous-year growth on current year increment reported above). We furthermore assumed a recovery of the effect to begin in the third growing season after the wind event. The perturbation dummies for the first and third growing season after storm were thus set to 0.6 and 0.85, respectively. To study the sensitivity of results to this perturbation pattern we additionally conducted a set of interrupted time series analyses varying first and third year perturbation dummies between 0.5 and 1.0 at increments of 0.1 (total of 216 unique combinations). Our analysis was restricted to the first three growing seasons post storm, as sufficient growth data beyond 2007 were not available. To control for the effects of a second storm that hit parts of the study area in 2007, we introduced a second, separate perturbation coefficient for this year in the respective counties, in order to separate the effect of this second perturbation from the one of the 2005 storm Gudrun analyzed here. Fitted perturbation coefficients, i.e., the wind effect on tree growth determined by the interrupted ARIMA models, were analyzed for significance, and related to data on wind speed as well as structural storm damage [3]. It is important to note that since the literature reports both positive and negative growth changes in response to strong winds at the level of individual trees [11]–[18] all analyses evaluating net effect at the county scale were conducted as two-sided tests.

### Upscaling of functional wind effects

In order to quantify the overall functional effect of Gudrun at the landscape scale and relate it to structural damage we scaled the relative growth changes determined in the ARIMA analysis up to absolute values (i.e., m^3^) for the 6.79·million ha study landscape. This upscaling was facilitated by basing our analyses on tree cores of the NFI, i.e., on tree records from a representative sample of southern Swedish Norway spruce forests. We extracted the 2005–2007 growth estimate for Norway spruce >60 years per county group from the NFI database (*i_abs_*), and estimated absolute wind-induced growth changes (*w_abs_*) from relative growth changes (*w_rel_*, i.e., ARIMA perturbation coefficients) according to Eq. 1:
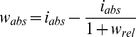
(1)All analyses were conducted using the R Project for Statistical Computing v2.13.1 [55], particularly applying the library forecast [56] for ARIMA modeling.
